# Relation of Cannabis Use Frequency and Gambling Behavior in Individuals Who Gamble Under the Influence of Cannabis

**DOI:** 10.1007/s10899-025-10381-3

**Published:** 2025-03-03

**Authors:** Abby McPhail, James P. Whelan, Meredith K. Ginley, Rory A. Pfund

**Affiliations:** 1 Tennessee Institute for Gambling Education & Research, 400 Fogelman Drive, Memphis, TN 38152 USA; 2 Department of Psychology, University of Memphis, Memphis, TN 38152 USA; 3 Department of Psychology, East Tennessee State University, 420 Rogers-Stout Hall, Johnson City, TN 37614 USA

## Abstract

There appears to be a significant positive relation between problematic cannabis use and problem gambling behaviors. Recent reviews have noted that individuals who use cannabis more frequently may experience less acute executive functioning impairment than those who use cannabis less often. The current study explored the relation between cannabis use frequency and problem gambling outcomes in those who gamble under the influence of cannabis, to explore if increased cannabis use frequency increases reported gambling problems, or is the reported effect on their gambling behavior is lessened in individuals who consume cannabis regularly? 769 individuals who gambled at least weekly were recruited from a crowdsource platform. These individuals reported their gambling behavior and cannabis use. To explore the relation between cannabis use frequency and problem gambling severity, regression models following both a simple linear model and a quadratic model were generated and evaluated for model fit and significance. The quadratic model was found to best fit the relation between cannabis use frequency and problem gambling severity. The quadratic model was also found to best fit the relation between frequency of time spent gambling under the influence of cannabis and problem gambling severity. Those who consumed cannabis infrequently or very frequently reported fewer gambling problems overall compared to those who consumed cannabis at a moderate frequency. The acute relation between cannabis use and gambling may be more complex than simply amplifying problematic gambling behaviors.

Cannabis is currently the third most popular drug worldwide (Connor et al., 2021); as cannabis becomes increasingly accessible, questions about cannabis’ effects on behavior become more important to address, especially cannabis’ effects on risk taking. Gambling is one area where the effects of cannabis on gambling behavior need to be assessed in order to prevent harm (Winters & Whelan, 2020).

Gambling is risking something of value on an outcome that is partially determined by chance (Whelan et al., 2007) and is legally accessible throughout much of the world (Abbott, 2020). Most who gamble do not experience harms, but some develop problematic gambling or a diagnosis of gambling disorder (American Psychiatric Association, 2013). The consequences of gambling disorder or problems include financial, personal, psychological, and social harms. Prevalence of problem gambling was estimated to be around 1.41% of the adult population (Tran et al., 2024) with the greatest harm found in marginalized groups (Abbott, 2020).

Research has found a significant relation between problematic cannabis use and problem gambling behaviors. One representative national study found that one-third of individuals with a cannabis use disorder were also experiencing gambling problems (Barnes et al., 2015). Several researchers have found that problematic cannabis use and problem gambling have shared risk factors (Barnes et al., 2015) and similar demographic profiles (Grant et al., 2015; Kerridge et al., 2018). This evidence provides a compelling argument for a high co-occurrence between cannabis use and gambling. However, this body of research is limited by a lack of studies empirically examining the acute effects of gambling under the influence of cannabis (Punia et al., 2021).

In a survey about concurrent engagement in cannabis and gambling, 805 individuals who gambled at least weekly were asked about their gambling and cannabis use. 30% endorsed gambling under the influence of cannabis (GUIC) at least some of the time (McPhail et al., 2020). Those who reported GUIC gambled more frequently than those who did not. In a similar study of at least monthly gamblers (*N* = 10,054), 56.2% of cannabis users reported GUIC (McGrath et al., 2023). This study also found that cannabis use was generally associated with both higher problem gambling severity and greater engagement in most forms of gambling. These studies support that GUIC is common amongst gamblers. Considering the potential negative cognitive effects of cannabis on gambling decision-making (Winters & Whelan, 2020), further exploration would be valuable.

Evidence into the acute effects of cannabis on risk taking has shown mixed findings. In executive functioning throughout areas relating to gambling, cannabis has been shown to produce acute short-term impairment of learning and memory (Schoeler & Bhattacharyya, 2013). Frequent cannabis use, but not chronic use, may impair attention and concentration (Hart et al., 2001; Morrison et al., 2009). Experimental studies using risk-taking tasks have also found mixed results; some found no significant effects of THC on risk-taking behavior (Ramaekers et al., 2006; McDonald et al., 2003), while Lane et al. (2005) found that participants given a high dose of THC engaged in significantly greater risk-taking behavior than those receiving a low dose or placebo. These documented neurological effects lead to questions about the behavioral impact of cannabis use while gambling in non-experimental settings.

Consistent obstacles that have been highlighted in assessing the acute effects of cannabis usage on gambling include a lack of quality research in the area (Dellazizzo et al., 2022), and difficulties in accounting for recurrent usage, which could produce mixed results due to unaccounted for tolerance (Cohen & Weinstein, 2018), or residual effects of cannabis that was previously consumed (Dellazizzo et al., 2022). One review concluded that cannabis use has small to moderate acute negative effects on executive functioning, but noted that an individual’s overall cannabis use pattern moderates these effects - individuals who use cannabis more frequently are less impaired overall (Dellazizzo et al., 2022). A second review noted that while results are mixed, those who use cannabis regularly were less likely to be impaired by cannabis than those who do not (Colizzi & Bhattacharyya, 2018). The authors proposed that the relation between use and impairment could be due to the development of a form of tolerance. This proposed effect requires further research to be better understood, however researchers have suggested potential neurobiological or behavioral adaptations such as pharmodynamic tolerance, learned compensatory behaviors, or increased volitional control (see Ramaekers et al., 2020).

The current study aims to explore the relation between cannabis use variables and problem gambling outcomes in those who gamble under the influence of cannabis. Does increased cannabis use frequency amplify gambling problems, or do individuals who gamble under the influence of cannabis report less severe gambling behavior when they consume cannabis regularly? To explore this relation, the relation between cannabis use variables and outcomes on a problem gambling assessment will be examined in both linear and non-linear models.

## Materials and Methods

### Participants

These participants were part of a larger study examining past year gambling experiences of individuals who gamble at least weekly. Respondents had to be residing in the U.S., be at least 18 years old, and report gambling at least once per week. Recruited via Amazon Mechanical Turk (MTurk), prospective participants were required both to have at least an 80% Human Intelligence Task approval rate (i.e. they have completed tasks on MTurk previously and have had their completed responses approved by requesters in at least 80% of tasks) and to pass a CAPCHA test to be eligible. Eligibility requirements were met by 802. After data cleaning and removing inconsistent responders, the sample size was 769. The mean age was 36.86 (*SD* = 10.83). Most identified as male (65.6%), white (69.2%), heterosexual (88.2%), and held bachelor’s degrees or higher (72%). Over half were married (59.2%). Table 1 displays demographics for this sample.

**Table 1:** Sample demographics for whole sample, for those who gamble under the influence of cannabis (GUIC) and those who do not GUIC

Categorical variables		Total Sample (*N* = 769)	No GUIC (*n* = 414)	GUIC (*n* = 355)
Gender				
	Male	505	257	248
	Female	261	156	105
Race/Ethnicity				
	White	532	307	225
	Other	237	107	130
Sexual Orientation				
	Heterosexual	678	385	293
	Not Exclusively Heterosexual	91	29	62
Education				
	No College	57	40	17
	Some College	158	106	52
	Batchelor’s Degree or Above	554	268	286
Marital Status				
	Married	455	196	118
	Other	314	218	237
Income				
	Less than $30,000	105	49	56
	$30,000 - $59,999	260	122	138
	$ 60,000 - $89,999	204	108	96
	$ 90,000 or greater	200	135	65

### Measures

#### Demographics

Participants reported their age, gender, race/ethnicity, sexual orientation, relationship status, if they have children in their household, income, and education.

#### Gambling Behavior

Three items assessed participants’ number of gambling days per week, time spent gambling, and money spent on each gambling day.

#### Problem Gambling Severity Index (PGSI)

The 9-item PGSI assessed participants’ gambling behaviors over the past year (Ferris & Wynne, 2001). Responses were indicated on a 4-point Likert type scale, ranging from 0 = *not at all* to 3 = *often*, with summed item ratings yielding a maximum score of 27. PGSI scores classify individuals as being at no risk (PGSI = 0), low-risk (PGSI = 1–2), moderate-risk (PGSI = 3–7), or high risk (PGSI ≥ 8) for experiencing gambling problems. These scores correlated with clinical interviews (*r* =.48, Ferris & Wynne, 2001).

#### Cannabis Use

One item assessed frequency of general cannabis use, asking participants, “Over the past year, how many days per month did you typically consume cannabis?”.

#### Cannabis and Gambling

Two items assessed cannabis use while gambling. One asked, “What percent of the time you gambled were you also under the influence of cannabis in the past year?” A second item asked, “During the last year, during a typical session when you gambled under the influence of cannabis, how high were you?” Response options were: “not at all high,” “a little bit high,” “moderately high,” “very high” and “extremely high.” This question and response options were taken from the Daily Sessions, Frequency, Age of Onset and Quantity of Cannabis Use Inventory (Cuttler & Spradlin, 2017).

## Procedure

Formal ethics approval for this study was granted by the University’s Institutional Review Board (IRB ID: PRO-FY2020-455, date of approval: 04.10.2020). After affirming informed consent, participants completed a series of eligibility questions. Eligible participants were asked to complete a questionnaire packet, including the measures described above and other questions about gambling and risk behaviors. It took approximately 40 min to complete all materials. Lastly, participants were provided with contact information and websites for finding mental health resources and problem gambling treatment services. They then received their reimbursement code.

## Data Analytic Approach

All analyses were completed using Statistical Package for the Social Sciences (SPSS), version 26. Eight participants were removed for being extreme univariate outliers in age (SD exceeded +/-3.29). Twenty-five participants were removed for inconsistent responses. Inconsistent responses were identified by questions that were asked repeatedly and answered differently. For example, participants were asked how many days per week they gambled as part of the inclusion questionnaire and then asked again during the main study. For all other continuous variables, outliers were identified by percentiles and were replaced with the trimmed mean. No more than 20% of data points for any single variable were replaced following guidance from Downey and King (1998).

To explore the relation between GUIC and gambling outcomes, independent samples t-tests were used. Participants who reported 1% or higher of their gambling time was spent GUIC were classified as part of the “GUIC” group, those who reported 0% were classified in the “no-GUIC” group. To explore the relation between cannabis variables (i.e., cannabis use frequency, percentage of gambling time spent GUIC, how high a participant reported being while gambling) and total PGSI score, three regression models that evaluated both a simple linear model and a non-linear quadratic model were generated and evaluated for model fit and significance.

## Results

### Descriptive Statistics

As can be seen in Table 2, the 769 individuals gambled 3.85 (*SD* = 1.92) days per week, risked an average of US$256.62 (*SD* = $276.91) per gambling session, and gambled an average of 4.89 (*SD* = 4.13) hours per gambling day. As expected, almost half (48.24%; *n* = 371) met the PGSI categorization as a problem gambler, with an average PGSI score of 7.61 (*SD* = 5.79) (see Table 2). Among those who reported no cannabis use, 27.4% (*n* = 103) were classified as problem gamblers. Comparatively, 68.2% (*n* = 268) of individuals who endorsed cannabis use were classified as problem gamblers. The Cronbach’s alpha of the PGSI in the total sample was 0.913, within the GUIC group α = 0.881, and within the no GUIC group α = 0.901, this indicates high levels of internal consistency of PGSI scores across items in our total sample and our comparison groups.

**Table 2:** T-test comparisons of gambling behavior variables between those who gamble under the influence of cannabis (GUIC) and those who do not GUIC

Gambling variable	Total Sample (*N* = 769) M(SD)	No GUIC (*n* = 414) M(SD)	GUIC (*n* = 355) M(SD)	T	*p*
Percent of gambling time spent gambling under the influence of cannabis			46.39(29.62)		
Amount risked on average gambling day ($)	256.62 (276.91)	200.11 (252.86)	322.53 (289.27)	6.26	< 0.001
Number of hours gambling on a gambling day	4.89 (4.13)	3.19 (2.65)	6.87 (4.64)	13.73	< 0.001
Gambling days per week	3.85 (1.92)	3.52 (2.01)	4.22 (1.76)	5.12	< 0.001
Types of gambling engaged in	9.13 (6.23)	5.91 (4.87)	12.88 (5.95)	18.60	< 0.001
PGSI score	7.61 (5.79)	5.17 (4.87)	10.45 (5.47)	14.56	< 0.001

Within this sample, 44% (*n* = 345) of individuals endorsed GUIC. Those who GUIC reported using cannabis an average of 15.10 (*SD* = 8.79) days per month and reported being under the influence of cannabis an average of 46.39% (*SD* = 29.62) of their total gambling time. When asked how high they were during a typical session where they GUIC, 8.2% reported “not at all high,” 34.1% reported “a little bit high,” 36.9% reported “moderately high,” 15.5% reported “very high” and 5.4% reported “extremely high”.

There were significant differences between those who do and do not GUIC on all gambling behavior variables (see Table 2). Individuals who reported GUIC risked more money, gambled for more hours, gambled more frequently, and engaged in a wider range of gambling types than those who did not gamble under the influence of cannabis (all *ps <* 0.001; Table 2). Notably, a higher proportion of those who GUIC were identified as problem gamblers (68.2%, *n* = 268) and reported significantly higher scores overall.

### Cannabis Use Frequency

**Fig. 1:**
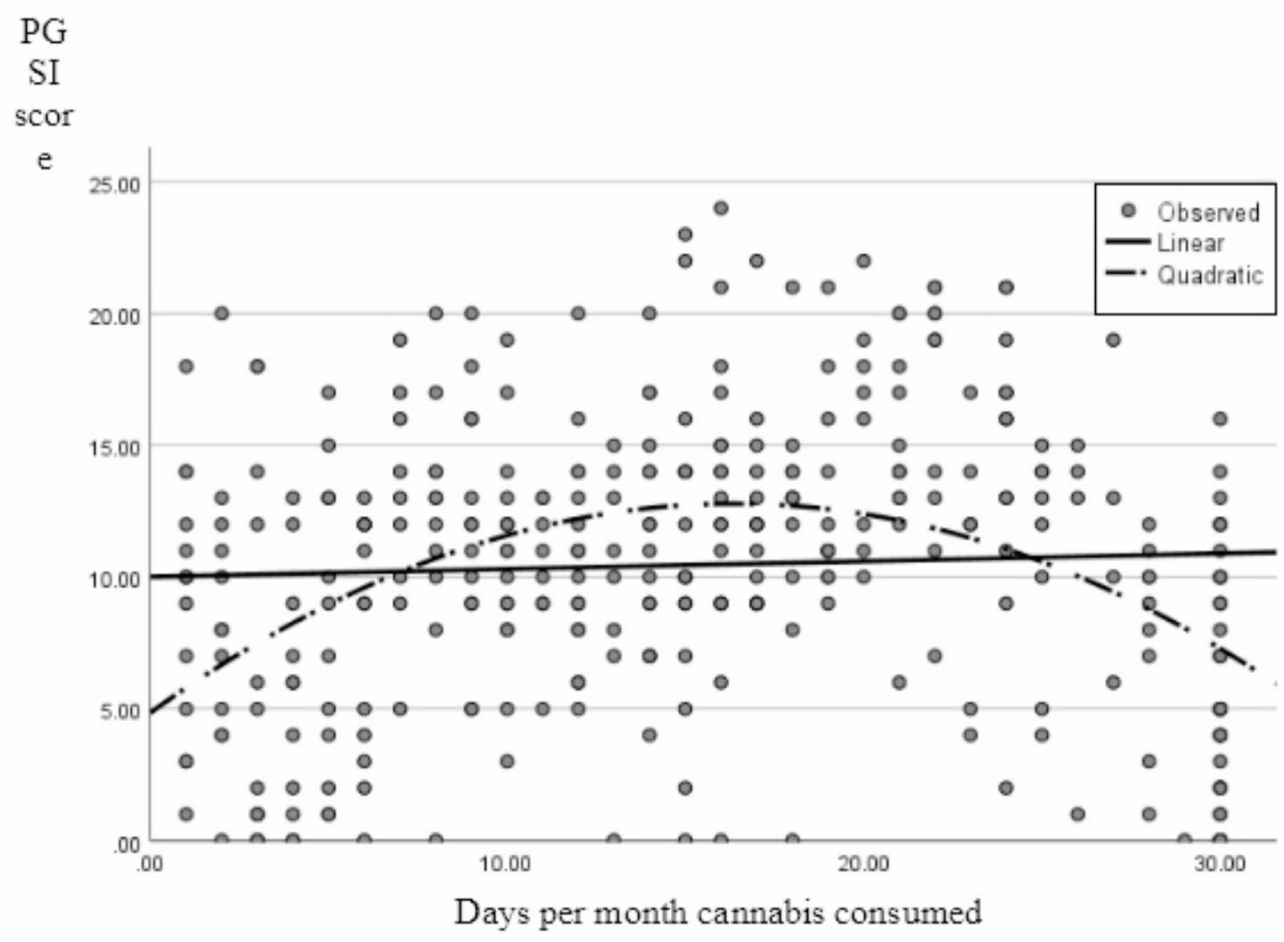
Linear and quadratic regression lines with cannabis use frequency as a predictor for PGSI score

Linear and quadratic regression models were computed predicting PGSI score from cannabis use frequency. The linear regression model was non-significant *r*^*2*^ = 0.002, *F* (1,352) = 23.79, *p* =.374. The addition of the quadratic term produced a significant curvilinear model *r*^*2*^ = 0.165, *F* (2,351) = 34.69, *p* <.001. Within the quadratic model, both the linear and quadratic cannabis use variables significantly predicted PGSI score (both *p* <.001; Table 3) and *r*^*2*^ increased by 0.163, supporting a quadratic model was a better fit. As seen in Fig. 1, these data were represented by an inverted U-shaped curve, with those who reported cannabis use the least or most days of the month scoring lower on the PGSI than those who reported moderate frequency of use.

**Table 3:** A comparison between linear, and quadratic models of the relationship between cannabis variables and scores on the problem gambling severity index (PGSI)

Model	b1	b2	F	*p*	*R* ^2^	*R*^2^ Change
Cannabis use frequency predictor model						
Linear	0.03		0.79	0.374	0.002	0.002
Quadratic	0.97**	-0.03**	34.69	< 0.001	0.165	0.163
*Percent of gambling time under the influence of cannabis predictor model*						
Linear	0.04**		13.97	< 0.001	0.038	0.038
Quadratic	0.27**	-0.02**	38.34	< 0.001	0.179	0.141
*How high were you while gambling predictor model*						
Linear	2.02**		53.32	< 0.001	0.132	0.132
Quadratic	3.64*	-0.28	27.52	< 0.001	0.136	0.004

### Percent of Gambling Time Spent Under the Influence of Cannabis

**Fig. 2:**
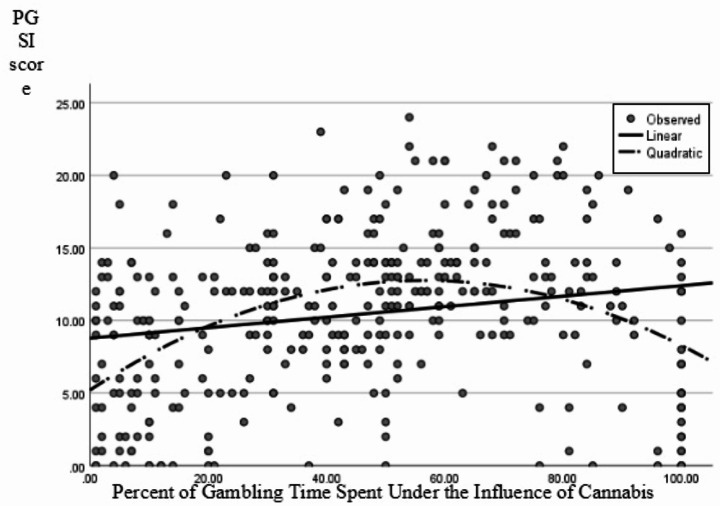
Linear and quadratic regression lines with percentage of gambling time spent under the influence of cannabis as a predictor for PGSI score

Linear and quadratic regression models were also tested and compared on model fit with percentage of gambling time spent GUIC acting as a predictor for PGSI score. The linear regression model was significant *r*^*2*^ = 0.038, *F* (1,352) = 13.96, *p* = < 0.001, as was the quadratic model *r*^*2*^ = 0.179, *F* (2,351) = 38.34, *p* <.001. The quadratic model explained higher variance overall with *r*^2^ increasing by 0.141 and both the quadratic and linear variables were significant within the quadratic model (Table 3); thus, the quadratic model was judged to better fit the data overall. The relation, as shown in Fig. 2, was represented by an inverted U-shaped curve with PGSI score increasing as percentage of time spent GUIC increased until a point where those who spent the largest proportion of their gambling time GUIC scored lower on the PGSI.

### How High Were You when You GUIC?

The how high variable was found to have a significant linear relation with PGSI scores, *r*^*2*^ = 0.129, *F* (1,352) = 53.32, *p* <.001. The quadratic model was also significant overall, *r*^*2*^= 0.136, *F* (2,351) = 27.52, *p* <.001, however the quadratic variable was not significant (*p* =.203, Table 3) and the *r*^*2*^ difference was 0.007. Therefore, the linear model was retained (Fig. 1).

## Discussion

In the current study, we examined the relation between cannabis use variables and problematic gambling behavior in a retrospective, self-report survey of individuals who gamble frequently. We found a significant quadratic relation between the frequency of cannabis use and risk of problem gambling. This relation indicated that those who consumed cannabis the least and the most were more likely to report a lower risk of problem gambling behaviors than those who consumed cannabis at a moderate frequency. When exploring the relation between the percentage of gambling time spent GUIC with risk of problem gambling, we also found a significant quadratic relation. This relation indicated that those who spent low or high proportions of their gambling time GUIC reported lower risk of problem gambling than those who spent a moderate amount of their gambling time GUIC.

These results indicate that the relation between acute cannabis consumption and gambling may be more complex than previous studies suggest, warranting further exploration. Our main finding was the non-linear relation between the frequency of general cannabis use and problem gambling score in frequent gamblers. Previous findings into individuals who gamble under the influence of cannabis reported a linear effect of cannabis use increasing negative gambling behavior without testing non-linear models (McGrath et al., 2023; McPhail et al., 2020). Exploring both linear and non-linear models suggested that those who consumed cannabis infrequently or very frequently had fewer self-reported gambling problems overall than those who consumed cannabis at a moderate frequency. A similar curvilinear relation was revealed between the percentage of time spent GUIC and level of gambling problems. Spending a larger proportion of gambling time GUIC increased gambling problems until a certain point whereby spending the majority or all of gambling time GUIC decreased problem gambling. These findings were consistent with the proposal of a potential behavioral adaptation or tolerance of cannabis use effects on cognitive functioning. For example, individuals who engaged in more frequent general cannabis use may have either experienced less adverse effects of cannabis on cognition or experienced more accurate expectations of how cannabis use would affect their behavior and potentially adapted their behavior accordingly (Ramaekers et al., 2020).

One explanation could be that individuals’ prior expectations about experiences before engaging in behavior have been found to impact behavior (Jones et al., 2001). Research into expectations around substance use’s effects on gambling behavior has shown that those who consume alcohol and gamble frequently tend to hold an overly positive view of how alcohol affects their gambling behavior (Horn et al., 2023). Research into how individuals expect cannabis to influence gambling has found that individuals expect that cannabis use will improve their gambling experience or lead to more positive outcomes (e.g., more likely to win) of their gambling behaviors (Smith et al., 2024). It has been suggested that individuals who use cannabis frequently but experience less behavioral impairment may have more accurate expectations of how cannabis affects their functioning and may then alter their behaviors accordingly (Anthenien et al., 2021; Dellazzio et al., 2022). This suggestion has previously been used to explain differences in memory outcomes between frequent and infrequent cannabis users, where frequent users were less impaired than those who used less often when completing a memory test after consuming the same amount of cannabis (Schoeler & Bhattacharyya, 2013). Testing this expectancy hypothesis would require examining this relation among those who were known to differ in the frequency of cannabis consumption.

In contrast, how high an individual reported being while gambling appeared to be better explained as linearly related to problem gambling outcomes. This linear relation indicates that consuming cannabis more frequently while gambling is related to more negative outcomes. Viewing this issue through the expectancy lens (Bandura, 1977), it could be that individuals who consume cannabis and gamble to a high level of intoxication cannot alter their behavior from past knowledge because they are too intoxicated.

A potential limitation of this study is that this sample utilized a survey approach rather than an experimental approach. Despite the importance of understanding the effect of cannabis use on gambling behavior, there are legal and ethical challenges to bring data to bear on this effect. Regulations on cannabis research limit experimental studies where individuals consume cannabis in the United States. Thus, studies exploring the effects of cannabis on gambling behavior have primarily depended on a survey approach. However, asking people to recall behavioral history can increase measurement error, particularly if asked to recall behavior over a long period of time (Serre et al., 2015). Relatedly, this study did not collect specifics of cannabis consumption from participants, and we did not ask about the potency of the cannabis consumed or the method of consumption participants used. These factors can impact the levels of intoxication experienced and should be examined in more detail in future research.

A second limitation was that data was collected through a crowdsourced convenience sample, which may have biased results (Mellis & Bickel, 2020). Samples collected using Mturk tend to include respondents who are younger, employed less than full-time, and more politically liberal than the general U.S. population (Goodman & Paolacci, 2017). However, recent studies have suggested that Mturk may be useful for recruiting samples of people who regularly gamble and is acceptable for sampling those who use cannabis (Kim & Hodgins, 2017). Relatedly, we chose to collect an adult sample of individuals aged 18 and over. As the legal age of gambling in the U.S. is 21, even in states where both gambling and cannabis use are legal, we were asking individuals to report their own engagement in an illegal behavior, possibly resulted in some individuals under-reporting. Additionally in this study we did not consider the legal status of cannabis use or gambling as a potential covariate as we felt this was beyond the scope of the current study. However, this may limit the generalizability of our findings.

A third limitation was that the current study did not identify the cannabis use frequencies in the curvilinear relation between cannabis use frequency and gambling problems. In other words, defining more precisely what levels of cannabis consumption led to reduced gambling problems experienced. Future research should include a sensitivity analysis to explore this effect further.

In future research the relation between cannabis use and gambling needs to be explored by including a more thorough examination of cannabis usage generally, outside of gambling, and while gambling by including different variables as indicators of cannabis usage, and a diagnostic measure. Potential future research would also benefit from moving away from the limitations of a retrospective self-report study design. A future study utilizing an experimental design could provide further information on this relation by collecting a sample of participants with different levels of general cannabis use frequency and recording their behavior while gambling under the influence of cannabis in a lab setting. Experimental studies relating to cannabis use while gambling can be challenging due to legal issues. A second methodology that a future study may use to further explore this relation should be an ecological momentary assessment or diary study where participants are tracked over a period of time, and record their gambling and cannabis use multiple times a day. Future studies in this area utilizing different methodologies are needed to more fully understand this relation.

## Conclusions

This study suggests that we need to consider more complex models in understanding cannabis’ acute effects on gambling behavior. We should be considering and evaluating an individual’s general cannabis use and be aware that individuals who consume cannabis frequently may experience less acute impairment. These findings have potential implications for the clinical treatment of individuals who use cannabis heavily while engaging in gambling behavior. Clinicians must make attempts to assess how clients’ cannabis usage may be impacting their gambling behavior while considering their broader cannabis use patterns, rather than only focusing on their simultaneous cannabis and gambling behavior. These findings also have potential policy or regulatory implications, in the U.S. at present, it is illegal to consume cannabis on non-tribal casino properties, as cannabis is illegal federally. However, if cannabis is federally legalized then the industry will have to decide how to regulate cannabis use while gambling, and responsible gambling messaging will be needed on GUIC to prevent harm. Further research into how gambling under the influence of cannabis affects gambling is necessary, to inform this guidance.

## Data Availability

The data that supported this study are available from the corresponding author, AM, upon reasonable request.

## References

[CR1] Abbott, M. (2020). The changing epidemiology of gambling disorder and gambling-related harm: Public health implications. *Public Health*, *184*. 10.1016/j.puhe.2020.04.003 10.1016/j.puhe.2020.04.003 32402593

[CR2] American Psychiatric Association. (2013). *Diagnostic and statistical manual of mental disorders* (5th ed., p. 223). American Psychiatric Association.

[CR3] Anthenien, A., Prince, M., Wallace, G., Jenzer, T., & Neighbors, C. (2021). Cannabis outcome expectancies, Cannabis use motives, and Cannabis use among a small sample of frequent using adults. *Cannabis*, *4*(1), Article1. 10.26828/cannabis/2021.01.005 PMC10212271 37287995

[CR4] Bandura, A. (1977). Social learning theory. *Social Learning Theory.*, viii, 247–viii, 247.

[CR5] Barnes, G. M., Welte, J. W., Tidwell, M. C. O., & Hoffman, J. H. (2015). Gambling and substance use: Co-occurrence among adults in a recent general population study in the united States. *International Gambling Studies*, *15*(1), 55–71. 10.1080/14459795.2014.990396 25914605 10.1080/14459795.2014.990396 PMC4405260

[CR6] Cohen, K., & Weinstein, A. (2018). The effects of cannabinoids on executive functions: Evidence from Cannabis and synthetic cannabinoids—A systematic review. *Brain Sciences*, *8*(3), 40. 10.3390/brainsci8030040 29495540 10.3390/brainsci8030040 PMC5870358

[CR7] Colizzi, M., & Bhattacharyya, S. (2018). Cannabis use and the development of tolerance: A systematic review of human evidence. *Neuroscience and Biobehavioral Reviews*, *93*, 1–25. 10.1016/j.neubiorev.2018.07.014 30056176 10.1016/j.neubiorev.2018.07.014

[CR8] Connor, J. P., Stjepanović, D., Le Foll, B., Hoch, E., Budney, A. J., & Hall, W. D. (2021). Cannabis use and cannabis use disorder. *Nature Reviews Disease Primers*, *7*(1), 16. 10.1038/s41572-021-00247-4 33627670 10.1038/s41572-021-00247-4 PMC8655458

[CR9] Cuttler, C., & Spradlin, A. (2017). Measuring cannabis consumption: Psychometric properties of the daily sessions, frequency, age of onset, and quantity of Cannabis use inventory (DFAQ-CU). *Plos One*, *12*(5), e0178194. 10.1371/journal.pone.0178194 28552942 10.1371/journal.pone.0178194 PMC5446174

[CR10] Dellazizzo, L., Potvin, S., Giguère, S., & Dumais, A. (2022). Evidence on the acute and residual neurocognitive effects of cannabis use in adolescents and adults: A systematic meta-review of meta‐analyses. *Addiction*, *117*(7), 1857–1870. 10.1111/add.15764 35048456 10.1111/add.15764

[CR11] Downey, R. G., & King, C. V. (1998). Missing data in likert ratings: A comparison of replacement methods. *Journal of General Psychology*, *125*(2), 175–191. 10.1080/00221309809595542 9935342 10.1080/00221309809595542

[CR12] Ferris, J., & Wynne, H. (2001). *The Canadian Problem Gambling Index: Final Report*.

[CR13] Goodman, J. K., & Paolacci, G. (2017). Crowdsourcing consumer research. *Journal of Consumer Research*, *44*(1), 196–210. 10.1093/jcr/ucx047

[CR14] Grant, B. F., Goldstein, R. B., Saha, T. D., Chou, S. P., Jung, J., Zhang, H., Pickering, R. P., Ruan, W. J., Smith, S. M., Huang, B., & Hasin, D. S. (2015). Epidemiology of DSM-5 alcohol use disorder: Results from the National epidemiologic survey on alcohol and related conditions. *JAMA Psychiatry*, *72*(8), 757–766. 10.1001/jamapsychiatry.2015.0584 26039070 10.1001/jamapsychiatry.2015.0584 PMC5240584

[CR15] Hart, C. L., van Gorp, W., Haney, M., Foltin, R. W., & Fischman, M. W. (2001). Effects of acute smoked marijuana on complex cognitive performance. *Neuropsychopharmacology: Official Publication of the American College of Neuropsychopharmacology*, *25*(5), 757–765. 10.1016/S0893-133X(01)00273-1 11682259 10.1016/S0893-133X(01)00273-1

[CR16] Horn, T. L., Lerma, M., Pfund, R. A., & Whelan, J. P. (2023). Expectations about how alcohol consumption influences gambling. *International Gambling Studies*, *0*(0), 1–14. 10.1080/14459795.2023.2224858

[CR17] Jones, B. T., Corbin, W., & Fromme, K. (2001). A review of expectancy theory and alcohol consumption. *Addiction*, *96*(1), 57–72. 10.1046/j.1360-0443.2001.961575.x 11177520 10.1046/j.1360-0443.2001.961575.x

[CR18] Kerridge, B. T., Pickering, R., Chou, P., Saha, T. D., & Hasin, D. S. (2018). DSM-5 cannabis use disorder in the National epidemiologic survey on alcohol and related Conditions-III: Gender-specific profiles. *Addictive Behaviors*, *76*, 52–60. 10.1016/j.addbeh.2017.07.012 28755613 10.1016/j.addbeh.2017.07.012

[CR19] Kim, H. S., & Hodgins, D. C. (2017). Reliability and validity of data obtained from alcohol, cannabis, and gambling populations on Amazon’s mechanical Turk. *Psychology of Addictive Behaviors*, *31*(1), 85–94. 10.1037/adb0000219 27893213 10.1037/adb0000219

[CR20] Lane, S. D., Cherek, D. R., Tcheremissine, O. V., Lieving, L. M., & Pietras, C. J. (2005). Acute marijuana effects on human risk taking. *Neuropsychopharmacology: Official Publication of the American College of Neuropsychopharmacology*, *30*(4), 800–809. 10.1038/sj.npp.1300620 15775958 10.1038/sj.npp.1300620

[CR21] McDonald, J., Schleifer, L., Richards, J. B., & de Wit, H. (2003). Effects of THC on behavioral measures of impulsivity in humans. *Neuropsychopharmacology: Official Publication of the American College of Neuropsychopharmacology*, *28*(7), 1356–1365. 10.1038/sj.npp.1300176 12784123 10.1038/sj.npp.1300176

[CR22] McGrath, D. S., Williams, R. J., Rothery, B., Belanger, Y. D., Christensen, D. R., el-Guebaly, N., Hodgins, D. C., Nicoll, F., Shaw, C. A., Smith, G. J., & Stevens, R. M. G. (2023). Problem gambling severity, gambling behavior, substance use, and mental health in gamblers who do and do not use cannabis: Evidence from a Canadian National sample. *Addictive Behaviors*, *137*, 107520. 10.1016/j.addbeh.2022.107520 36257248 10.1016/j.addbeh.2022.107520

[CR23] McPhail, A., Whelan, J. P., Peter, S. C., Li, Q., Winters, K. C., & Meyers, A. W. (2020). Sweetening the pot: Exploring differences between frequent gamblers who do and do not gamble under the influence of cannabis. *Addictive Behaviors*, *110*, 106531. 10.1016/j.addbeh.2020.106531 32682270 10.1016/j.addbeh.2020.106531

[CR24] Mellis, A. M., & Bickel, W. K. (2020). Mechanical Turk data collection in addiction research: Utility, concerns and best practices. *Addiction*, *115*(10), 1960–1968. 10.1111/add.15032 32135574 10.1111/add.15032 PMC7483427

[CR25] Morrison, P. D., Zois, V., McKeown, D. A., Lee, T. D., Holt, D. W., Powell, J. F., Kapur, S., & Murray, R. M. (2009). The acute effects of synthetic intravenous Delta9-tetrahydrocannabinol on psychosis, mood and cognitive functioning. *Psychological Medicine*, *39*(10), 1607–1616. 10.1017/S0033291709005522 19335936 10.1017/S0033291709005522

[CR26] Punia, K., DeVillaer, M., MacKillop, J., & Balodis, I. M. (2021). Understanding the overlap between Cannabis use and gambling behaviour: A systematic review of empirical findings and consideration of policy implications. *Current Addiction Reports*, *8*(1), 35–56. 10.1007/s40429-020-00323-x

[CR27] Ramaekers, J. G., Kauert, G., van Ruitenbeek, P., Theunissen, E. L., Schneider, E., & Moeller, M. R. (2006). High-potency marijuana impairs executive function and inhibitory motor control. *Neuropsychopharmacology: Official Publication of the American College of Neuropsychopharmacology*, *31*(10), 2296–2303. 10.1038/sj.npp.1301068 16572123 10.1038/sj.npp.1301068

[CR28] Ramaekers, J. G., Mason, N. L., & Theunissen, E. L. (2020). Blunted highs: Pharmacodynamic and behavioral models of cannabis tolerance. *European Neuropsychopharmacology*, *36*, 191–205. 10.1016/j.euroneuro.2020.01.006 32014378 10.1016/j.euroneuro.2020.01.006

[CR29] Schoeler, T., & Bhattacharyya, S. (2013). The effect of cannabis use on memory function: An update. *Substance Abuse and Rehabilitation*, *4*, 11–27. 10.2147/SAR.S25869 24648785 10.2147/SAR.S25869 PMC3931635

[CR30] Serre, F., Fatseas, M., Swendsen, J., & Auriacombe, M. (2015). Ecological momentary assessment in the investigation of craving and substance use in daily life: A systematic review. *Drug and Alcohol Dependence*, *148*, 1–20. 10.1016/j.drugalcdep.2014.12.024 25637078 10.1016/j.drugalcdep.2014.12.024

[CR31] Smith, E. H., McPhail, A., Lermas, M., Pfund, R. A., & Whelan, J. P. (2024). Expectations of How Acute Cannabis Use Affects Gambling Experiences and Behaviors. *Cannabis*. https://publications.sciences.ucf.edu/cannabis/index.php/Cannabis/article/view/231 10.26828/cannabis/2024/000231 PMC11225979 38975592

[CR32] Tran, L. T., Wardle, H., Colledge-Frisby, S., Taylor, S., Lynch, M., Rehm, J., Volberg, R., Marionneau, V., Saxena, S., Bunn, C., Farrell, M., & Degenhardt, L. (2024). The prevalence of gambling and problematic gambling: A systematic review and meta-analysis. *The Lancet Public Health*, *9*(8), e594–e613. 10.1016/S2468-2667(24)00126-9 39025095 10.1016/S2468-2667(24)00126-9

[CR33] Whelan, J. P., Meyers, A. W., & Steenbergh, T. A. (2007). *Problem and pathological gambling*. Hogrefe & Huber.

[CR34] Winters, K. C., & Whelan, J. P. (2020). Gambling and Cannabis use: Clinical and policy implications. *Journal of Gambling Studies*, *36*(1), 223–241. 10.1007/s10899-019-09919-z 31828696 10.1007/s10899-019-09919-z

